# Predictors of Influenza and Pneumococcal Vaccination Among Participants in the Women’s Health Initiative

**DOI:** 10.1177/00333549221081817

**Published:** 2022-03-18

**Authors:** Jonathan Fix, Macarius M. Donneyong, Stephen R. Rapp, Maryam Sattari, Beverly M. Snively, Jean Wactawski-Wende, Emily W. Gower

**Affiliations:** 1 Department of Epidemiology, Gillings School of Global Public Health, University of North Carolina at Chapel Hill, Chapel Hill, NC, USA; 2 Outcomes and Translational Sciences, College of Pharmacy, The Ohio State University, Columbus, OH, USA; 3 Health Services Management and Policy, College of Public Health, The Ohio State University, Columbus, OH, USA; 4 Department of Psychiatry and Behavioral Medicine, Wake Forest School of Medicine, Winston-Salem, NC, USA; 5 Department of Social Sciences and Health Policy, Wake Forest School of Medicine, Winston-Salem, NC, USA; 6 Department of Medicine, Division of General Internal Medicine, University of Florida College of Medicine, Gainesville, FL, USA; 7 Department of Biostatistics and Data Science, Division of Public Health Sciences, Wake Forest School of Medicine, Winston-Salem, NC, USA; 8 Department of Epidemiology and Environmental Health, School of Public Health and Health Professions, University at Buffalo, Buffalo, NY, USA

**Keywords:** vaccination, predictors, influenza, pneumococcal pneumonia, older adults

## Abstract

**Objective:**

Older adults typically experience higher rates of severe disease and mortality than the general population after contracting an infectious disease. Vaccination is critical for preventing disease and severe downstream outcomes; however, vaccination rates among older adults are suboptimal. We assessed predictors associated with pneumococcal and seasonal influenza vaccination among older women.

**Methods:**

We used data from the Women’s Health Initiative, a nationwide cohort of women. We ascertained seasonal influenza and pneumococcal vaccination status through a questionnaire administered in 2013. We limited analyses to women aged ≥65 years at questionnaire administration. We used logistic regression to estimate associations between demographic, lifestyle, and health-related factors and vaccination and explored stratification by race.

**Results:**

Of participants who responded to each question, 84.3% (n = 60 578) reported being vaccinated for influenza and 85.5% (n = 59 015) for pneumonia. The odds of reporting influenza vaccination were significantly lower among non-Hispanic Black participants than among non-Hispanic White participants (odds ratio [OR] = 0.53; 95% CI, 0.49-0.58), women with no health insurance versus private health insurance (OR = 0.61; 95% CI, 0.54-0.68), and women living in rural versus urban settings (OR = 0.84; 95% CI, 0.73-0.96). Current smoking, lower education levels, and having comorbid conditions were associated with lower likelihood of being vaccinated for influenza (than not); past pneumonia diagnosis and being currently married were associated with a higher likelihood. We observed similar associations for pneumococcal vaccination coverage.

**Conclusions:**

These findings reinforce the need to enact policy and implement programs to improve access to, education and awareness about, and provider recommendations for these critical disease-prevention tools. Results from our study should guide strategies for SARS-CoV-2 vaccination.

The Centers for Disease Control and Prevention (CDC) estimated that during the 2017-2018 influenza season, nearly 400 000 hospitalizations and 30 000 influenza-related deaths occurred.^1^ Although older adults accounted for only 16% of the population,^2^ they contributed 54.5% of influenza-related hospitalizations and 77% of influenza-related deaths.^1^ Similarly, older adults are most susceptible to pneumococcal disease complications, accounting for 60% of pneumococcal disease–related hospitalizations and 72% of deaths yearly.^3^

In the United States, vaccination rates for these diseases are suboptimal among older adults. In the 2019-2020 season, seasonal influenza vaccine coverage among people aged ≥65 years was 69.3%. Vaccination rates varied across demographic groups, with non-Hispanic Black and Hispanic people having had the lowest rates.^4^ Similarly, only 69.0% of women aged ≥65 years reported having received a pneumococcal vaccine in 2018, and proportions vaccinated varied by demographic characteristics.^5^

Identifying characteristics associated with likelihood of vaccination coverage is important for improving adherence with vaccination recommendations. Prior studies have explored sociodemographic factors and comorbidities associated with vaccine coverage among older adults.^5-10^ However, few studies have examined the relationship between lifestyle variables and vaccination. This study used data from the Women’s Health Initiative (WHI) to examine a broad range of potential predictors of vaccination among a cohort of women aged ≥65 years with a high rate of vaccination. This information should guide the development of targeted policies and programs to increase access to and awareness of essential vaccines for older women.

## Methods

### Study Population

The WHI is an ongoing longitudinal study, initiated in 1993, that enrolled about 161 000 postmenopausal women in the United States. The study initially included participants in either a clinical trial or an observational study. Although the interventional components have ended, the WHI continues to follow participants regularly. The WHI contacts participants via mail or telephone annually to complete questionnaires and provide updated health-related and sociodemographic information.^11^ During some annual contacts, the study administers supplemental questionnaires to capture additional information. We restricted the current analyses to participants who completed a 2013 supplemental questionnaire, the only questionnaire through which participants were asked to provide vaccination information. The institutional review board at the University of North Carolina at Chapel Hill determined this study to be exempt from continuing review.

### Ascertainment of Vaccination Status

The WHI’s 2013 supplemental questionnaire included 2 vaccination questions: (1) “During the past 12 months, have you had a seasonal flu shot?” and (2) “A pneumonia shot or pneumococcal vaccine is usually given only once or twice in a person’s life and is different from the flu shot. Have you ever had a pneumonia shot?” For both questions, participants could respond yes, no, or don’t know/not sure. Our primary analyses excluded those who responded with “don’t know/not sure.” In a secondary analysis, we considered this response as a discrete group. We restricted the population to women aged ≥65 years, per CDC’s Advisory Committee on Immunization Practices recommendation for universal pneumococcal pneumonia vaccination^12^ and to maintain consistency between the influenza and pneumonia vaccination analytic sets.

### Predictor Identification and Classification

We evaluated sociodemographic and health-related characteristics (Online-Only Supplementary Table 1). Briefly, we used self-reported baseline data for race and ethnicity, education, urbanicity, annual household income, marital status, and health insurance. Through a 2011 questionnaire, participants reported their current smoking status, alcohol usage, exercise history, and self-rated health. The 2013 supplemental questionnaire included questions about pneumonia history and use of the internet for health information. Information on key medical conditions was collected throughout the study by using updates to annual medical history.

We used participants’ 2010 zip code and the US Department of Agriculture’s Rural–Urban Commuting Area codes^13^ and accompanying classifications to categorize the urbanicity of participants’ residence. To assess chronic conditions, we grouped participant reports into the following: asthma and emphysema; dementia, Alzheimer disease, and Parkinson disease; diabetes, hypertension, and high cholesterol; and myocardial infarction, stroke, transient ischemic attack, deep venous thrombosis, and pulmonary embolism. We created these groups to combine diseases with similar etiology, morbidity, and health care utilization.

### Statistical Methods

A total of 74 852 WHI participants completed the vaccination history questionnaire. We excluded from further analyses 10 participants who were aged <65 years when they provided their vaccination history, resulting in a final analytic sample of 74 842. We calculated means and SDs for continuous variables and counts and percentages for categorical variables. We modeled influenza and pneumococcal vaccination separately, and we modeled the probabilities of reporting not being vaccinated by using logistic regression. In multivariable regression, we included all predictors that were significant in the univariate analysis at an α of ≤ .10. We performed 2 sets of secondary analyses. First, we further investigated participants who responded “don’t know/not sure” to the vaccination questions by evaluating the association between each potential predictor and vaccination status, with 3 possible outcomes—yes, no, and don’t know/missing—to determine whether the don’t-know group was similar to another group. Separately, we assessed predictors of vaccination coverage limited to those who self-classified as either non-Hispanic White or non-Hispanic Black to better understand race- and ethnic-specific risk and protective factors associated with vaccination. We conducted all statistical analyses in SAS version 9.4 (SAS Institute, Inc).

## Results

Of the 74 842 participants in our analysis, the mean age was 78.2 years (range, 65.4-98.0 y); 88.7% were non-Hispanic White, 46.8% had a college degree or higher, and 97.6% lived in an urban area or large rural city/town (Table 1).

**Table 1.** Characteristics of Women’s Health Initiative participants aged ≥65 years who responded to a supplemental questionnaire on vaccination and health, United States, 2013^a^

Characteristic	All questionnaire respondents (N = 74 842)	Influenza-vaccinated respondents^b^ (n = 60 578)	Pneumonia-vaccinated respondents^c^ (n = 59 015)
Mean age, y	78.2	78.3	78.2
Race and ethnicity			
Non-Hispanic White	88.7	89.8	90.0
Non-Hispanic Black	5.5	4.8	4.7
Hispanic	2.4	2.2	2.2
American Indian/Alaska Native	0.3	0.3	0.3
Asian/Pacific Islander^d^	2.1	2.1	2.0
Other^e^	0.9	0.8	0.9
Education			
Some high school or less	2.5	2.2	2.2
High school diploma/GED	14.9	14.7	14.5
School after high school	35.8	35.1	35.6
College degree or higher	46.8	48.0	47.7
Annual household income, $			
<10 000	2.0	1.8	1.8
10 000-34 999	28.7	27.7	28.0
35 000-74 999	44.6	44.8	44.9
≥75 000	24.8	25.8	25.4
Urbanicity^f^			
Urban or large rural city/town	97.6	97.7	97.7
Small rural town	2.4	2.3	2.3
Marital status			
Never married	4.1	4.0	4.0
Divorced or separated	14.8	13.9	14.3
Widowed	12.2	12.0	11.9
Currently married	67.0	68.3	68.0
Marriage-like relationship	1.9	1.8	1.8
Smoking status			
Current	2.1	1.9	2.0
Past	45.9	46.5	46.4
Never	52.0	41.6	51.7
Alcohol use, no. of times per week^g^			
Never	30.5	29.6	30.0
<1-4	52.7	53.1	53.1
≥5	16.8	17.3	16.9
Exercise, no. of days per week^h^			
0 or 1	59.3	58.7	58.9
2 or 3	27.6	28.2	28.0
≥4	13.1	13.1	13.1
Self-rated health			
Excellent	13.1	12.7	12.7
Very good	44.5	44.5	44.6
Good	34.7	35.0	35.0
Fair	7.3	7.3	7.3
Poor	0.5	0.5	0.5
Stayed at nursing home in past year	2.2	2.2	2.2
Use of internet for health information	58.9	59.7	60.7
Past pneumonia diagnosis	25.3	26.0	27.5
Health insurance			
None	3.5	3.0	3.1
Medicaid	0.6	0.5	0.5
Private, Medicare, military, or other	95.9	96.5	96.4
Chronic diseases^i^			
History of cancer (any)	17.3	17.8	17.8
Alzheimer disease, Parkinson disease, or dementia	5.6	5.4	4.9
Asthma or emphysema	13.1	13.3	13.8
Diabetes, hypertension, or high cholesterol	77.0	78.0	77.9
Myocardial infarction, stroke, transient ischemic attack, deep vein thrombosis, or pulmonary embolism	2.8	15.2	15.3
History of hip fracture	2.8	2.7	2.8

Abbreviation: GED, General Educational Development.

a Data source: The Women’s Health Initiative Study Group.^11^ All values are percentage, except age, which is presented as mean.

b A total of 71 858 participants responded to the question on influenza vaccination.

c A total of 69 041 participants responded to the question on pneumococcal vaccination.

d Indicated on the questionnaire as “Asian or Pacific Islander (ancestry is Chinese, Indo-Chinese, Korean, Japanese, Pacific Islander, Vietnamese).”

e Indicated on the questionnaire as a free-text response: “Other (specify): ___.”

f Participants’ 2010 zip code and the US Department of Agriculture’s Rural–Urban Commuting Area codes^13^ and accompanying classifications were used to categorize the urbanicity of participants’ residence.

g In response to the following question: “In the past 3 months, how often have you had drinks containing alcohol?”

h In response to the following question: “How often each week do you do moderate or strenuous exercise?”

i Number of positive (yes) responses to history of any of the listed chronic diseases.

### Influenza Vaccination

Overall, 60 578 (84.3%) of the 71 858 participants responding to the influenza vaccine question reported having received the vaccination in the 12 months before completing the questionnaire (Table 1). The univariable (Online-Only Supplementary Table 2) and multivariate (Table 2) analyses yielded similar results. In the multivariate analysis, vaccination increased with increasing age (odds ratio [OR] = 1.03 per year; 95% CI, 1.03-1.04), and non-Hispanic Black and American Indian/Alaska Native participants were less likely than non-Hispanic White participants to have received the influenza vaccine (OR = 0.53 [95% CI, 0.49-0.58] and OR = 0.61 [95% CI, 0.42-0.87], respectively). Participants with less education than a college degree (vs those with a college degree or higher) and those living in small rural towns (vs an urban area or large rural city/town) were less likely to be vaccinated (OR range = 0.72-0.83 and OR = 0.84 [95% CI, 0.73-0.96], respectively), while being currently married (vs other categories of marital status) was associated with the highest odds of vaccination.

**Table 2.** Multivariate models of predictors of influenza and pneumococcal pneumonia vaccination among Women’s Health Initiative participants aged ≥65 years who responded to a questionnaire on vaccination history, United States, 2013^a^

Characteristic	Influenza vaccination^b^		Pneumonia vaccination^c^	
	Proportion vaccinated, %	Odds ratio (95% CI)	Proportion vaccinated, %	Odds ratio (95% CI)
Age, y	NA	1.03 (1.03-1.04)	NA	1.04 (1.04-1.05)
Race and ethnicity				
Non Hispanic White	85.3	1 [Reference]	86.6	1 [Reference]
Non-Hispanic Black	72.8	0.53 (0.49-0.58)	73.6	0.50 (0.46-0.55)
Hispanic	77.5	0.88 (0.76-1.01)	76.8	0.74 (0.64-0.85)
American Indian/Alaska Native	76.4	0.61 (0.42-0.87)	81.7	0.80 (0.53-1.20)
Asian/Pacific Islander^d^	85.3	0.99 (0.84-1.16)	84.6	0.95 (0.80-1.12)
Other^e^	76.7	0.57 (0.46-0.71)	79.6	0.62 (0.50-0.78)
Education				
Some high school or less	77.3	0.72 (0.62-0.84)	77.9	0.65 (0.56-0.76)
High school diploma/GED	83.3	0.83 (0.77-0.89)	83.7	0.78 (0.72-0.84)
School after high school	82.8	0.78 (0.74-0.83)	84.9	0.85 (0.80-0.90)
College degree or higher	86.2	1 [Reference]	86.9	1 [Reference]
Annual household income, $				
<10 000	76.6	1 [Reference]	79.2	1 [Reference]
10 000-34 999	82.0	0.92 (0.78-1.09)	84.5	0.95 (0.80-1.14)
35 000-74 999	84.7	1.03 (0.87-1.22)	85.8	1.00 (0.83-1.20)
≥75 000	87.3	1.22 (0.74-1.46)	87.1	1.10 (0.91-1.33)
Urbanicity^f^				
Urban or large rural city/town	84.4	1 [Reference]	85.6	1 [Reference]
Small rural town	80.7	0.84 (0.73-0.96)	82.0	0.81 (0.70-0.93)
Marital status				
Never married	82.3	0.86 (0.77-0.97)	84.8	0.97 (0.85-1.10)
Divorced or separated	79.1	0.75 (0.70-0.80)	82.5	0.87 (0.81-0.93)
Widowed	84.0	0.84 (0.78-0.91)	84.9	0.80 (0.74-0.87)
Currently married	85.8	1 [Reference]	86.4	1 [Reference]
Marriage-like relationship	79.9	0.68 (0.58-0.79)	83.0	0.82 (0.70-0.97)
Smoking status				
Current	76.1	0.73 (0.64-0.84)	79.5	0.79 (0.68-0.92)
Past	85.5	1.11 (1.06-1.17)	86.3	1.05 (1.00-1.10)
Never	83.7	1 [Reference]	85.2	1 [Reference]
Alcohol use, times per week^g^				
Never	82.3	1 [Reference]	84.6	1 [Reference]
<1-4	84.7	1.12 (1.07-1.19)	85.8	1.04 (0.98-1.10)
≥5	86.9	1.23 (1.14-1.33)	86.5	1.00 (0.92-1.08)
Exercise, days per week^h^				
0 or 1	83.6	1 [Reference]	85.3	—
2 or 3	85.7	1.18 (1.11-1.24)	86.2	—
≥4	85.0	1.13 (1.05-1.22)	85.7	—
Self-rated health				
Excellent	81.6	1 [Reference]	82.2	1 [Reference]
Very good	83.8	1.15 (1.07-1.23)	85.0	1.17 (1.09-1.25)
Good	85.7	1.37 (1.27-1.48)	87.0	1.37 (1.27-1.48)
Fair	86.4	1.52 (1.35-1.71)	88.1	1.55 (1.37-1.76)
Poor	84.9	1.29 (0.91-1.84)	87.0	1.39 (0.94-2.05)
Use of internet for health information				
Yes	84.9	1.18 (1.12-1.24)	86.8	1.42 (1.35-1.50)
No	83.4	1 [Reference]	83.6	1 [Reference]
Past pneumonia diagnosis				
Yes	86.9	1.25 (1.18-1.32)	91.5	1.99 (1.87-2.13)
No	83.4	1 [Reference]	83.3	1 [Reference]
Health insurance				
None	70.5	0.61 (0.54-0.68)	74.1	0.62 (0.56-0.70)
Medicaid	78.6	0.79 (0.58-1.07)	80.8	0.79 (0.57-1.10)
Private, Medicare, military, or other	84.9	1 [Reference]	86.0	1 [Reference]
**Chronic diseases** ^i^				
Cancer (any)				
Yes	87.0	1.23 (1.16-1.32)	87.9	1.18 (1.10-1.26)
No	83.7	1 [Reference]	84.9	1 [Reference]
Alzheimer disease, Parkinson disease, or dementia				
Yes	84.8	—	81.2	0.63 (0.56-0.70)
No	84.3	—	85.7	1 [Reference]
Asthma or emphysema				
Yes	86.1	1.13 (1.05-1.21)	89.7	1.36 (1.26-1.48)
No	84.0	1 [Reference]	84.8	1 [Reference]
Diabetes, hypertension, or high cholesterol				
Yes	85.6	1.50 (1.43-1.59)	86.6	1.39 (1.32-1.47)
No	80.0	1 [Reference]	81.9	1 [Reference]
Myocardial infarction, stroke, transient ischemic attack, deep vein thrombosis, or pulmonary embolism				
Yes	85.7	0.98 (0.92-1.06)	87.6	1.04 (0.96-1.12)
No	84.1	1 [Reference]	85.1	1 [Reference]
History of hip fracture				
Yes	85.6	—	88.3	1.04 (0.88-1.24)
No	84.3	—	85.4	1 [Reference]

Abbreviations: —, variable not included in the multivariate model; GED, General Educational Development; NA, not applicable.

a Data source: The Women’s Health Initiative Study Group.^11^

b A total of 71 848 participants responded to the question on influenza vaccination.

c A total of 69 041 participants responded to the question on pneumococcal vaccination.

d Indicated on the questionnaire as “Asian or Pacific Islander (ancestry is Chinese, Indo-Chinese, Korean, Japanese, Pacific Islander, Vietnamese).”

e Indicated on the questionnaire as a free-text response: “Other (specify): ___.”

f Participants’ 2010 zip code and the US Department of Agriculture’s Rural–Urban Commuting Area codes^13^ and accompanying classifications were used to categorize the urbanicity of participants’ residence.

g In response to the following question: “In the past 3 months, how often have you had drinks containing alcohol?”

h In response to the following question: “How often each week do you do moderate or strenuous exercise?”

i Number of positive (yes) responses to history of any of the listed chronic diseases.

Current smokers were less likely than past or never smokers to be vaccinated. Participants who reported ≥1 alcoholic drink per week or exercising ≥2 days per week were more likely than never drinkers and those who exercised ≤1 day per week to be vaccinated. Participants who reported having no health insurance were less likely than those with any form of health insurance to have received the influenza vaccine (OR = 0.61; 95% CI, 0.54-0.68). Having reported a prior pneumonia diagnosis (OR = 1.25; 95% CI, 1.18-1.32), a history of cancer (OR = 1.23; 95% CI, 1.16-1.32), asthma or emphysema (OR = 1.13; 95% CI, 1.05-1.21), or diabetes, hypertension, or high cholesterol (OR = 1.50; 95% CI, 1.43-1.59) were associated with higher odds of vaccination, compared with not having reported these conditions.

### Pneumococcal Vaccination

Of the 69 041 participants who responded to the pneumococcal vaccination question, 59 015 (85.5%) reported having received the vaccination (Table 1).

In multivariable analysis, predictors associated with pneumococcal vaccination were similar to the observations for influenza vaccination (Table 2), with some notable exceptions. Hispanic participants were less likely than non-Hispanic White participants (OR = 0.74; 95% CI, 0.64-0.85) to be vaccinated, while the test of association for American Indian/Alaska Native participants was not significant. Past pneumonia diagnosis was more strongly associated with higher likelihood of pneumococcal vaccination (OR = 1.99; 95% CI, 1.87-2.13) than was observed for influenza vaccination. While not associated with influenza vaccination, reporting a history of Alzheimer disease, Parkinson disease, or dementia (vs not reporting a history of these conditions) was associated with a lower likelihood of being vaccinated for pneumonia (OR = 0.63; 95% CI, 0.56-0.70). In addition, we did not find significant associations in the comparisons of participants who were never married (vs currently married), by levels of weekly alcohol use or by exercise history.

### Secondary Analyses

Participants who responded “don’t know/not sure” to vaccination questions were small subsets of the total study population (2994 [4.0%] for influenza and 5801 [7.7%] for pneumonia). Their predictor distribution more closely resembled the distribution for participants who responded as vaccinated, compared with participants who responded as not vaccinated. Including them in the vaccinated group did not affect overall findings. Among participants who received an influenza vaccination, 91.6% were also vaccinated for pneumonia, compared with 52.3% among those who had not received an influenza vaccination.

In multivariable analyses restricted to non-Hispanic Black participants, some associations with vaccination remained similar to those identified in the whole cohort, including age, education, past pneumonia diagnosis, health insurance, cancer history, and several chronic diseases; however, other predictors were more highly associated with vaccination (Table 3). Living in a small rural town (vs in an urban area or large rural city/town) was associated with having 0.22 times the odds of being vaccinated for influenza (95% CI, 0.07-0.65). The magnitude of this association was nearly one-quarter the size of that among non-Hispanic White participants (OR = 0.86; 95% CI, 0.74-0.99). Reporting less than a high school diploma/General Educational Development (vs reporting a college degree or higher) was associated with being less than half as likely to be vaccinated against influenza (OR = 0.47; 95% CI, 0.32-0.69), while we found a much smaller difference when we compared higher levels of education. In the full cohort, each increase in education level was associated with a decreased risk of not being vaccinated. Some subpopulations had particularly low levels of vaccination; only 43.5% of non-Hispanic Black participants without health insurance and no comorbidities were vaccinated for influenza, compared with 67.8% of non-Hispanic White participants who similarly did not have health insurance or comorbidities, per unadjusted analysis.

**Table 3.** Univariate and multivariate models of predictors of influenza vaccination, stratified by race and ethnicity, among Women’s Health Initiative participants aged ≥65 years who responded to a questionnaire on vaccination history, United States, 2013^a^

Characteristic	Non-Hispanic Black (n = 3939)^b^			Non-Hispanic White (n = 63 638)^b^		
	No. of respondents (% vaccinated)	Univariate, OR (95% CI)	Multivariate, OR (95% CI)	No. of respondents (% vaccinated)	Univariate, OR (95% CI)	Multivariate, OR (95% CI)
Age, y	NA	1.03 (1.01-1.04)	1.03 (1.01-1.04)	NA	1.03 (1.03-1.04)	1.03 (1.03-1.04)
Education						
Some high school or less	203 (61.1)	0.53 (0.39-0.71)	0.47 (0.32-0.69)	1237 (80.8)	0.63 (0.54-0.72)	0.73 (0.61-0.88)
High school diploma/GED	450 (72.0)	0.86 (0.68-1.09)	0.79 (0.61-1.03)	9529 (84.1)	0.79 (0.74-0.84)	0.81 (0.75-0.87)
School after high school	1460 (72.7)	0.89 (0.76-1.05)	0.85 (0.71-1.02)	22 489 (83.8)	0.77 (0.74-0.81)	0.77 (0.73-0.82)
College degree or higher	1785 (74.9)	1 [Reference]	1 [Reference]	29 996 (87.0)	1 [Reference]	1 [Reference]
Annual household income, $						
<10 000	225 (70.7)	1 [Reference]	—	906 (78.2)	1 [Reference]	1 [Reference]
10 000-34 999	1294 (71.0)	1.02 (0.75-1.39)	—	16 893 (83.4)	1.40 (1.19-1.65)	1.01 (0.83-1.22)
35 000-74 999	1630 (74.1)	1.18 (0.87-1.61)	—	27 151 (85.6)	1.66 (1.41-1.95)	1.12 (0.92-1.37)
≥75 000	603 (76.6)	1.36 (0.96-1.92)	—	15 458 (87.7)	2.00 (1.70-2.36)	1.32 (1.08-1.63)
Urbanicity^c^						
Urban or large rural city/town	3923 (72.9)	1 [Reference]	1 [Reference]	61 986 (85.4)	1 [Reference]	1 [Reference]
Small rural town	16 (50.0)	0.37 (0.14-0.99)	0.22 (0.07-0.65)	1658 (81.3)	0.74 (0.65-0.84)	0.86 (0.74-0.99)
Marital status						
Never married	242 (71.1)	0.80 (0.59-1.07)	0.84 (0.60-1.17)	2493 (84.0)	0.82 (0.74-0.92)	0.87 (0.76-0.99)
Divorced or separated	1230 (69.0)	0.72 (0.61-0.85)	0.73 (0.61-0.87)	8690 (80.9)	0.66 (0.62-0.70)	0.74 (0.69-0.80)
Widowed	619 (75.3)	0.99 (0.80-1.22)	0.87 (0.68-1.12)	7499 (85.0)	0.89 (0.83-0.95)	0.83 (0.76-0.90)
Currently married	1764 (75.5)	1 [Reference]	1 [Reference]	43 537 (86.5)	1 [Reference]	1 [Reference]
Marriage-like relationship	57 (63.2)	0.56 (0.32-0.96)	0.59 (0.32-1.09)	1232 (80.9)	0.67 (0.58-0.77)	0.66 (0.56-0.77)
Smoking status						
Current	152 (66.5)	0.76 (0.54-1.09)	—	1199 (77.2)	0.62 (0.54-0.71)	0.70 (0.60-0.82)
Past	1664 (74.0)	1.10 (0.95-1.28)	—	27 991 (86.4)	1.16 (1.10-1.21)	1.12 (1.07-1.18)
Never	1753 (72.2)	1 [Reference]	—	30 895 (84.6)	1 [Reference]	1 [Reference]
Alcohol use, times per week^d^						
Never	1753 (71.8)	1 [Reference]	—	17 064 (83.6)	1 [Reference]	1 [Reference]
<1-4	1693 (73.7)	1.10 (0.95-1.28)	—	32 385 (85.6)	1.16 (1.10-1.22)	1.14 (1.07-1.21)
≥5	145 (73.8)	1.10 (0.75-1.62)	—	10 959 (87.2)	1.34 (1.25-1.43)	1.24 (1.15-1.35)
Exercise, days per week^e^						
0 or 1	2194 (71.3)	1 [Reference]	1 [Reference]	35 330 (84.6)	1 [Reference]	1 [Reference]
2 or 3	989 (75.4)	1.23 (1.04-1.47)	1.33 (1.11-1.61)	16 585 (86.6)	1.18 (1.12-1.24)	1.17 (1.10-1.24)
≥4	354 (73.7)	1.13 (0.87-1.45)	1.30 (0.99-1.72)	7808 (85.8)	1.34 (1.02-1.17)	1.13 (1.05-1.23)
Self-rated health						
Excellent	239 (69.0)	1 [Reference]	—	8269 (82.3)	1 [Reference]	1 [Reference]
Very good	1256 (72.1)	1.15 (0.86-1.56)	—	27 416 (84.6)	1.18 (1.11-1.26)	1.16 (1.07-1.24)
Good	1672 (73.2)	1.23 (0.91-1.65)	—	20 167 (87.0)	1.45 (1.35-1.55)	1.40 (1.29-1.52)
Fair	390 (75.4)	1.37 (0.96-1.97)	—	4039 (87.9)	1.56 (1.40-1.75)	1.55 (1.36-1.77)
Poor	24 (79.2)	1.70 (0.61-4.74)	—	277 (85.9)	1.32 (0.93-1.86)	1.27 (0.86-1.87)
Use of internet for health information						
Yes	2056 (73.2)	1.04 (0.90-1.19)	—	37 770 (85.8)	1.09 (1.05-1.14)	1.18 (1.11-1.24)
No	1828 (72.4)	1 [Reference]	—	25 180 (84.6)	1 [Reference]	1 [Reference]
Past pneumonia diagnosis						
Yes	744 (79.3)	1.55 (1.28-1.88)	1.59 (1.27-1.98)	16 247 (87.4)	1.27 (1.21-1.34)	1.24 (1.17-1.32)
No	3126 (71.2)	1 [Reference]	1 [Reference]	46 148 (84.5)	1 [Reference]	1 [Reference]
Health insurance						
None	273 (65.9)	0.68 (0.53-0.89)	0.88 (0.64-1.20)	1940 (71.4)	0.41 (0.37-0.46)	0.56 (0.50-0.64)
Medicaid	74 (66.2)	0.69 (0.42-1.12)	0.71 (0.41-1.24)	278 (82.0)	0.76 (0.56-1.03)	0.81 (0.56-1.18)
Private, Medicare, military, or other	3523 (73.9)	1 [Reference]	1 [Reference]	61 047 (85.8)	1 [Reference]	1 [Reference]
**Chronic diseases** ^f^						
Cancer (any)						
Yes	504 (76.4)	1.24 (1.00-1.54)	1.17 (0.92-1.49)	10 817 (87.8)	1.29 (1.21-1.37)	1.25 (1.17-1.34)
No	3275 (72.4)	1 [Reference]	1 [Reference]	50 208 (84.8)	1 [Reference]	1 [Reference]
Alzheimer disease, Parkinson disease, or dementia						
Yes	212 (75.5)	1.16 (0.84-1.59)	—	3413 (86.0)	1.06 (0.96-1.17)	—
No	3727 (72.7)	1 [Reference]	—	60 225 (85.3)	1 [Reference]	—
Asthma or emphysema						
Yes	666 (77.0)	1.30 (1.05-1.60)	1.23 (0.97-1.56)	8190 (87.0)	1.18 (1.10-1.26)	1.13 (1.04-1.22)
No	3373 (72.1)	1 [Reference]	1 [Reference]	55 448 (85.1)	1 [Reference]	1 [Reference]
Diabetes, hypertension, or high cholesterol						
Yes	3544 (74.2)	1.83 (1.48-2.27)	2.01 (1.58-2.56)	48 272 (86.8)	1.56 (1.48-1.63)	1.50 (1.41-1.58)
No	395 (61.0)	1 [Reference]	1 [Reference]	15 366 (80.8)	1 [Reference]	1 [Reference]
Myocardial infarction, stroke, transient ischemic attack, deep vein thrombosis, or pulmonary embolism						
Yes	589 (75.0)	1.14 (0.93-1.40)	—	9687 (86.6)	1.13 (1.06-1.20)	0.98 (0.91-1.06)
No	3350 (72.5)	1 [Reference]	—	53 951 (85.1)	1 [Reference]	1 [Reference]
History of hip fracture						
Yes	25 (72.0)	0.96 (0.40-2.30)	—	1827 (86.0)	1.06 (0.93-1.21)	—
No	3895 (72.8)	1 [Reference]	—	61 611 (85.3)	1 [Reference]	—

Abbreviations: —, variable not included in multivariate model; GED, General Educational Development; NA, not applicable; OR, odds ratio.

a Data source: The Women’s Health Initiative Study Group.^11^

b Among participants who answered yes or no to the question on influenza vaccination. Number of respondents in each category (eg, education) may not sum to the value in the column head because not all participants answered all questions on characteristics.

c Participants’ 2010 zip code and the US Department of Agriculture’s Rural–Urban Commuting Area codes^13^ and accompanying classifications were used to categorize the urbanicity of participants’ residence.

d In response to the following question: “In the past 3 months, how often have you had drinks containing alcohol?”

e In response to the following question: “How often each week do you do moderate or strenuous exercise?”

f Number of positive (yes) responses to history of any of the listed chronic diseases.

## Discussion

This study identified key demographic (race and ethnicity, health insurance type, age, marital status, urbanicity, and education) and health-related (smoking, alcohol use, exercise history, self-rated health, prior pneumonia diagnosis, and chronic conditions) factors that were significantly associated with self-reported vaccination status among a population of women aged ≥65 years with a high rate of vaccination. These findings support the existing literature on predictors of vaccination and provide new insights into additional characteristics that may increase the risk of older women receiving influenza or pneumococcal vaccines. Our stratified analyses prompt interesting considerations regarding cohort design, namely, ensuring representation of key populations in nationwide cohorts.

Race was among the strongest predictors of vaccination in our study. A previous systematic review also reported that non-Hispanic Black and Hispanic people were less likely than non-Hispanic White people to be vaccinated,^7^ in part because of limited access to health care and lower levels of health insurance.^14^ Improving vaccination rates among racial and ethnic minority populations is critical, because US Black populations, compared with non-Black populations, have higher levels of comorbidities associated with more severe pneumococcal disease, longer lengths of stay, and higher costs during pneumococcal hospitalizations.^15^ We also found that living in small rural towns (vs urban areas or a large rural city/town) was associated with lower levels of vaccination, likely reflecting more limited health care access. This finding is consistent with a health insurance claim–based study of older adults indicating that older adults living in rural areas were 23% less likely than those in suburban/metropolitan areas to receive pneumococcal vaccination.^16^

Increasing access for key populations, such as racial and ethnic minority groups and individuals living in rural settings, could be achieved through developing partnerships with community groups, leaders, or nonprofit organizations. Efforts to improve influenza vaccination rates among hard-to-reach populations have benefited from working with churches in areas with high levels of participation and trust in faith-based centers.^17^ Research also has shown mobile health clinics to be highly effective at increasing health services, largely because of their patient-centric approach.^18^ Community members attending mobile health clinics cite their convenient locations, informal settings, and familiar environments as key aspects of increasing their engagement. Strategic partnerships with community organizations should be made to most effectively increase vaccination rates among older adults.

### Factors Involving Health Care Utilization or Health History

This study highlights that factors that increase the interaction with the health care system are associated with greater likelihood of vaccination. Our finding that participants with chronic diseases were more likely than participants without chronic diseases to be vaccinated is consistent with the existing literature.^8,19,20^ This finding may simply reflect that people in worse health have more interaction with the health care system than do people with better health; research indicates that people who report seeking medical care 1 or more times per year are more likely than people with no medical care visits in the previous 12 months to receive recommended vaccines.^21^ However, it may also show that individuals who are at higher risk of infection and progression to severe disease take proactive preventive measures more commonly than healthier individuals do. Indeed, we found that participants who reported a prior pneumonia diagnosis were substantially more likely than participants without a prior pneumonia diagnosis to report pneumococcal vaccination, potentially reflecting the importance of understanding disease severity and personal risk in vaccination decisions. A meta-analysis^22^ of risk perception and health behavior found that individuals who perceived themselves at higher risk of influenza were more likely to be vaccinated than individuals who perceived a lower risk. When exploring reasons for pneumococcal vaccination refusal among older Black patients, researchers found that those who refused vaccination may not have believed they were susceptible to the disease.^23^ This finding reinforces the importance of increased knowledge about vaccines and the diseases they protect against.

It is important to identify new strategies to promote routine and proactive interaction with the health care system. Direct outreach by primary care providers and pharmacists, through mailings or automated telephone calls, has increased the rates of influenza vaccination^24^ and human papillomavirus vaccine initiation and completion.^25^ Previous studies suggest that health care provider recommendation is the most influential factor contributing to a patient’s decision to vaccinate.^26-28^ Thus, it is critical that we maximize health care providers’ ability to recommend vaccination. This maximization can be accomplished by using new technologies such as electronic reminder systems,^29^ addressing financial barriers physicians face that limit their recommendations, and providing vaccines in primary care settings.^30,31^

The results of our race-and-ethnicity–stratified analysis reveal the importance of careful cohort design and participant recruitment. Stratification by race and ethnicity or other predictors can provide insights into factors affecting vaccination or other health outcomes. The presence of statistical significance for 1 subpopulation and absence for another may not necessarily indicate that the association is modified by variables used for stratification but instead that the study has greater statistical power to identify signals (whether clinically relevant or not) in one group over another. The impact of differential statistical power can be observed in the unadjusted associations between a history of myocardial infarction, stroke, transient ischemic attack, deep vein thrombosis, or pulmonary embolism and influenza vaccination. Despite the point estimate of the association among non-Hispanic Black participants being of greater magnitude (OR = 1.15) than that among non-Hispanic White participants (OR = 1.13), only the association among non-Hispanic White participants was significant. Future researchers should consider representation and sample size during cohort design and analysis.

### Potential Implications for SARS-CoV-2 Vaccination Strategies

Our data provide insights that could be leveraged to improve uptake of the SARS-CoV-2 vaccine. The original strain of SARS-CoV-2 had a reproductive number of roughly 2.5, corresponding to a herd protection threshold of about 60% vaccinated; however, the emergence and predominance of the Delta variant, which has a reproductive number of nearly 7, requires slightly more than 85% vaccination to achieve herd protection.^32^ More recently, the Omicron variant was estimated to be 4 times as infectious as the Delta variant.^33^ Recent analyses of patterns of intention to receive the COVID-19 vaccine^34^ and real-world data on vaccine administration in the United States^35^ are similar to patterns observed for the predictors of influenza and pneumococcal pneumonia vaccination assessed in our analysis. Lifestyle predictors of SARS-CoV-2 vaccine uptake have yet to be explored and would provide valuable information for further developing vaccination promotion programs. Consensus is growing in the scientific community that SARS-CoV-2 is likely to become endemic in the United States.^36^ If routine vaccination against SARS-CoV-2 is required in the future, public health officials should leverage lessons learned from influenza to increase the effectiveness and efficiency of programs to promote vaccination.

### Limitations and Strengths

Although to our knowledge this research represents the largest long-standing, prospective cohort study to examine vaccination among women aged ≥65 years, our study had some limitations. First, the vaccination data were collected in 2013, and behaviors may have changed since then. Second, we classified predictors by using self-reported information from the nearest time to which vaccination status was assessed. Although we used the most updated information possible, not all data on predictors were collected concurrently with data on vaccination status. Thus, our risk-factor categorization may not have reflected some participants’ true status at the time of questionnaire completion. Reliance on self-reported vaccination status may present an additional limitation, because the accuracy of this information may be associated with race and ethnicity, education, and household income.^37,38^ Third, our study population was mostly non-Hispanic White, educated, and living in urban settings, which may limit the generalizability of our overall findings. While adult women (aged ≥18 years) in the United States are more likely than adult men to get vaccinated for influenza, the differences by sex become smaller or even disappear completely with age.^4,7,39^ We could not find sex-stratified analyses of predictors of vaccination in the literature; this information would be helpful to better understand the effect of sex on vaccination and the generalizability of our findings. Nonetheless, the predictors identified here should assist researchers when they develop analysis plans for investigating vaccine uptake among other populations. Fourth, a higher proportion of participants reported having received influenza (84.3%) and pneumococcal (85.5%) vaccinations in comparison with the general population of older adults in the United States (70.4% and 66.9%, respectively). While acknowledging high rates of vaccination, our study reveals key predictors of vaccination among a specific population, which enables more efficient targeting to further increase vaccination levels.

Key strengths of our study included the use of a large, prospective cohort of women with detailed health information; leveraging demographic, health, and lifestyle information to complete a robust analysis of predictors of vaccination; and stratification by race and ethnicity to evaluate how predictors of vaccination differ by racial and ethnic groups. Assessing modification of predictors of vaccination by key demographic variables, such as race and ethnicity and urbanicity, in future research would enhance targeted efforts to increase vaccination among specific populations. Future research should also investigate the impact of health-related and lifestyle factors on vaccination among men and younger populations.

## Conclusions

Even among a group of women with high vaccination rates, we identified subgroups at higher risk of missed seasonal influenza and/or pneumococcal vaccination. This information adds to the growing body of literature about characteristics associated with vaccine coverage and should be used to craft interventions and programs aimed at improving vaccination coverage among older women. This information is directly applicable to SARS-CoV-2 vaccine distribution, given that we saw similar characteristics among one-time and annual vaccines. Clinicians working with older subpopulations with a higher risk of being unvaccinated should be more vigilant in recommending vaccinations during clinic visits.

## Supplemental Material

sj-docx-1-phr-10.1177_00333549221081817 – Supplemental material for Predictors of Influenza and Pneumococcal Vaccination Among Participants in the Women’s Health Initiative Click here for additional data file. Supplemental material, sj-docx-1-phr-10.1177_00333549221081817 for Predictors of Influenza and Pneumococcal Vaccination Among Participants in the Women’s Health Initiative by Jonathan Fix, Macarius M. Donneyong, Stephen R. Rapp, Maryam Sattari, Beverly M. Snively, Jean Wactawski-Wende and Emily W. Gower in Public Health Reports

sj-docx-2-phr-10.1177_00333549221081817 – Supplemental material for Predictors of Influenza and Pneumococcal Vaccination Among Participants in the Women’s Health Initiative Click here for additional data file. Supplemental material, sj-docx-2-phr-10.1177_00333549221081817 for Predictors of Influenza and Pneumococcal Vaccination Among Participants in the Women’s Health Initiative by Jonathan Fix, Macarius M. Donneyong, Stephen R. Rapp, Maryam Sattari, Beverly M. Snively, Jean Wactawski-Wende and Emily W. Gower in Public Health Reports
