# Supplementary material for: Predictors of Influenza and Pneumococcal Vaccination Among Participants in the Women’s Health Initiative
Source: Public Health Rep. 2022 Mar 18;138(2):281–91. doi: 10.1177/00333549221081817 (PMC10031837; doi:10.1177/00333549221081817)
Supplement: sj-docx-1-phr-10.1177_00333549221081817 – Supplemental material for Predictors of Influenza and Pneumococcal Vaccination Among Participants in the Women’s Health Initiative [file sj-docx-1-phr-10.1177_00333549221081817.docx]

**Online-Only Supplementary Table 1.** Variable collection and classification methods for study on predictors of influenza and pneumococcal vaccination among participants in the Women’s Health Initiative, 2013

| **Variable** | **Form(s) used** | **Question and variable responses** | **Method to update (year up to date)** | **Notes** |
| --- | --- | --- | --- | --- |
| Seasonal flu vaccination status | Form 156, Supplemental Questionnaire | During the past 12 months, have you had a seasonal flu shot?   - Yes - No - Don’t know/not sure | NA (2013) | Study outcome. Used exactly as recorded. |
| Pneumococcal pneumonia vaccination status | Form 156, Supplemental Questionnaire | A pneumonia shot or pneumococcal vaccine is usually given only once or twice in a person’s life and is different from the flu shot. Have you ever had a pneumonia shot?   - Yes - No - Don’t know/not sure | NA (2013) | Study outcome. Used exactly as recorded. |
| Age | Form 2, Eligibility Screening | What is your age now?   - Whole number response | Age at study entry plus years enrolled at time supplement was completed | Age at study entry variable + years since study entry − 0.5 years to reflect the midpoint of the previous year from the date the supplement was completed. |
| Race and ethnicity | Form 2, Eligibility Screening | How would you describe your racial or ethnic group? If you are of mixed blood, which group do you identify with most?   - American Indian or Alaskan Native - Asian or Pacific Islander - Black or African American (not of Hispanic origin) - Hispanic/Latino - White (not of Hispanic origin) - Other (specify) | NA, baseline (1993-1998) | Used exactly as recorded. |
| Highest education | Form 2, Eligibility Screening | What is the highest grade in school you finished?   - Didn’t go to school - Grade school (1-4 years) - Grade school (5-8 years) - Grade school (9-11 years) - High school diploma or GED - Vocational or training school after high school graduation - Some college or associate degree - College graduate or baccalaureate degree - Some college or professional school after college graduation - Master’s degree - Doctoral degree | NA, baseline (1993-1998) | Collapsed groups to feature new categories of:   - Some high school or less - High school diploma/GED - School after high school - College degree or higher |
| Annual household income | Form 2, Eligibility Screening | What was the total family income (before taxes) from all sources within your household in the last year?   - Less than $10,000 - $10,000 to $19,999 - $20,000 to $34,999 - $35,000 to $49,999 - $50,000 to $74,999 - $75,000 to $99,999 - $100,000 to $149,999 - $150,000 or more - Don’t know | NA, baseline (1993-1998) | Collapsed groups to feature new categories of:   - Less than $10,000 - $10,000 to $34,999 - $35,000 to $74,999 - $75,000 or more |
| Urbanicity index | Form 2, Eligibility Screening | What is your current mailing address?   - Zip | NA, used participant addresses on file at end of core study (2010) | Used census tract Federal Information Processing Standard (FIPS) codes that corresponded with geocoded addresses.  Participants were expected to provide updated addresses when they moved. |
| Marital status | Form 20, Personal Information | What is your current marital status?   - Never married - Divorced or separated - Widowed - Presently married - Living in a marriage-like relationship | NA, baseline (1993-1998) | Used exactly as recorded. |
| Smoking status | Form 155, Lifestyle Questionnaire (2011)  Form 34, Personal Habits (1993-1998) | Do you smoke cigarettes now?   - Yes - No   During your entire life, have you smoked at least 100 cigarettes?   - Yes - No | NA, year completed the Lifestyle Questionnaire (2011) | Smoking status was recorded as “current smoker” if the participant reported current smoking on their 2011 questionnaire, “past” if they reported “no” to current smoking and “yes” to having smoked at least 100 cigarettes in their lifetime on Form 34 at baseline, and “never” if they responded “no” to both current and ever smoking. |
| Alcohol use | Form 155, Lifestyle Questionnaire | In the past 3 months, how often have you had drinks containing alcohol?   - Never - Less than 1 per week - 1 or 2 times per week - 3 or 4 times per week - 5 or 6 times per week - Every day | NA, year completed the Lifestyle Questionnaire (2011) | Used exactly as recorded. |
| Exercise history | Form 155, Lifestyle Questionnaire | How often each week do you do moderate or strenuous exercise?   - 0 to 1 days per week - 2 days per week - 3 days per week - 4 days per week - 5 or more days per week | NA, year completed the Lifestyle Questionnaire (2011) | Collapsed groups to feature new categories of:   - 0 to 1 days per week - 2 days per week - 3 days per week - 4 or more days per week |
| Self-rated health | Form 155, Lifestyle Questionnaire | In general, would you say your health is:   - Excellent - Very good - Good - Fair - Poor | NA, year completed the Lifestyle Questionnaire (2011) | Used exactly as recorded. |
| Stayed at nursing home in past year | Form 155, Lifestyle Questionnaire | In the past year, have you stayed in a nursing home?   - No - Yes | NA, year completed the Lifestyle Questionnaire (2011) | Used exactly as recorded. |
| Use of internet for health information | Form 156, Supplemental Questionnaire | Do you use the internet to look for health information?   - No - Yes | NA (2013) | Used exactly as recorded. |
| Past pneumonia diagnosis | Form 156, Supplemental Questionnaire | As an adult, have you had pneumonia diagnosed by a physician?   - Yes - No - Don’t know/not sure | NA (2013) | Used exactly as recorded. |
| Health insurance | Form 20, Personal Information | Which category or categories below best describe how you usually pay for your medical care? (Mark all that apply.)   - Prepaid private insurance - Other private insurance - Medicare - Medicaid - Military or Veterans Administration-sponsored - No insurance - Other | NA, baseline (1993-1998) | Collapsed groups to feature new categories of:   - No insurance - Medicaid - Private, Medicare, military, or other insurance |
| History of cancer (any) | Form 33, Medical History Update (annual completion) | Since the date on the front of this form, has a doctor or other health care provider told you that you have a new cancer, malignant growth, or tumor? Do not include benign tumors.   - Yes - No | Any report of event on medical history update through date of supplement completion (2013) | First occurrence of any cancer, excluding non-melanoma skin cancer.  Adjudicated for all participants through supplement completion. Recorded as:   - Yes - No |
| Asthma or emphysema | Form 30, Medical History  Form 143, OS Year 3  Form 144, OS Year 4  Form 145, OS Year 5  Form 146, OS Year 6  Form 147, OS Year 7  Form 148, OS Year 8 | — | Specific to each chronic disease, see rows below. | Any positive (“yes”) response to history of asthma or emphysema. |
| Asthma | Form 30, Medical History  Form 143, OS Year 3  Form 144, OS Year 4  Form 145, OS Year 5  Form 146, OS Year 6  Form 147, OS Year 7  Form 148, OS Year 8 | In the past year, has a doctor told you that you have any of the following conditions? (19.3 asthma)   - Yes - No | CT – NA, baseline (1993-1998)  OS – latest data available up to OS Year 8 (1993-2003) | Self-reported asthma, not adjudicated. Recorded as:   - Yes - No |
| Emphysema | Form 30, Medical History  Form 143, OS Year 3  Form 144, OS Year 4  Form 145, OS Year 5  Form 146, OS Year 6  Form 147, OS Year 7  Form 148, OS Year 8 | In the past year, has a doctor told you that you have any of the following conditions? (19.4 emphysema)   - Yes - No | CT – NA, baseline (1993-1998)  OS – latest data available up to OS Year 8 (1993-2003) | Self-reported emphysema, not adjudicated. Recorded as:   - Yes - No |
| Dementia, Alzheimer’s disease, or Parkinson’s disease | Form 30, Medical History  Form 33, Medical History Update | — | Specific to each chronic disease, see rows below. | Any positive (“yes”) response to history of dementia, Alzheimer’s, or Parkinson’s |
| Dementia or Alzheimer’s disease | Form 30, Medical History  Form 33, Medical History Update (annual completion) | Since the date on the front of this form, has a doctor or other health care provider told you that you have any of the following conditions? Mark all that apply.   - Dementia or Alzheimer’s | Any report of event on medical history update through date of supplement completion (2013) | Self-report of Alzheimer’s during extensions 1 and 2. Nonadjudicated for all participants. Record as:   - Yes - No |
| Parkinson’s disease | Form 30, Medical History  Form 33, Medical History Update (annual completion) | Since the date on the front of this form, has a doctor or other health care provider told you that you have any of the following conditions? Mark all that apply.   - Parkinson’s disease | Any report of event on medical history update through date of supplement completion (2013) | Self-report of Alzheimer’s during extensions 1 and 2. Nonadjudicated for all participants. Recorded as:   - Yes - No |
| Diabetes, hypertension, or high cholesterol | Form 30, Medical History  Form 33, Medical History Update | — | Specific to each chronic disease, see rows below. | Any positive (“yes”) response to history of diabetes, hypertension, or hypercholesterolemia. |
| Diabetes | Form 30, Medical History  Form 33, Medical History Update (once in 2005, and annual during extension 2 through 2013) | Did a doctor ever say that you had sugar diabetes or high blood sugar when you were not pregnant?   - Yes - No   Since the date on the front of this form, has a doctor prescribed for the first time any of the following pills or treatments? (Mark all that apply.)   - Pills for diabetes - Insulin shots for diabetes | Doctor diagnoses recorded at beginning of extension 1 (2005), OR self-report of having treated diabetes with pills or insulin recorded through the date of supplement completion (2013) | Outcome defined as doctor diagnoses of ever having diabetes or high blood sugar at beginning of extension 1 OR self-report of having treated diabetes with pills or insulin through date of supplement completion:   - Yes - No |
| Hypertension | Form 30, Medical History  Form 33, Medical History Update (annual completion) | Did a doctor ever say that you had hypertension or high blood pressure? (Do not include high blood pressure that you had only when you were pregnant.)   - Yes - No   Since the date on the front of this form, has a doctor prescribed for the first time any of the following pills or treatments? (Mark all that apply.)   - Pills for high blood pressure or hypertension | Any report of diagnosed hypertension was recorded at baseline (1993-1998) OR any report of taking pills for hypertension through date of supplement completion (2013) | Outcome defined as any report of diagnosed hypertension at baseline OR any report of taking pills for hypertension through date of supplement completion:   - Yes - No |
| High cholesterol | Form 30, Medical History  Form 33, Medical History Update (annual completion during extension 1 through 2010) | Since the date on the front of this form, has a doctor prescribed for the first time any of the following pills or treatments? (Mark all that apply.)   - Pills for high cholesterol | Any self-report of high cholesterol requiring pills at baseline (1993-1998) OR self-report (both CT and OS) of taking cholesterol pills through extension 1 (2010) | Outcome was defined as any self-report of high cholesterol requiring pills at baseline OR self-report (both CT and OS) of taking cholesterol pills through extension 1:   - Yes - No |
| Myocardial infarction, stroke, transient ischemic attack, deep vein thrombosis, or pulmonary embolism | Form 30, Medical History  Form 33, Medical History Update | — | Specific to each chronic disease, see rows below. | Number of positive (“yes”) responses to history of the following: myocardial infarction, stroke, transient ischemic attack, deep vein thrombosis, or pulmonary embolism. |
| Myocardial infarction | Form 30, Medical History  Form 33, Medical History Update (annual completion) | Since the date on the front of this form, have you been diagnosed or treated for any of the following conditions or procedures? (myocardial infarction, heart attack [coronary, myocardial infarction])   - Yes - No | Any report of event on medical history update through date of supplement completion (2013) | Core study: Adjudicated for all.  Extension 1: Adjudicated for all.  Extension 2: Adjudicated for MRC; self-report for all others.  Used as recorded. |
| Stroke | Form 30, Medical History  Form 33, Medical History Update (annual completion) | Since the date on the front of this form, have you been diagnosed or treated for any of the following conditions or procedures? (Stroke)   - Yes - No | Any report of event on medical history update through date of supplement completion (2013) | Core study: Adjudicated for all.  Extension 1: Adjudicated for all.  Extension 2: Adjudicated for MRC; self-report for all others.  Used as recorded. |
| Transient ischemic attack | Form 30, Medical History  Form 33, Medical History Update (annual completion) | Since the date on the front of this form, has a doctor or other health care provider told you that you have any of the following conditions? Mark all that apply.   - Transient ischemic attack (not a stroke) | Any report of event on medical history update through date of supplement completion (2013) | Core study: Adjudicated for all.  Extension 1: Self-reported.  Extension 2: Adjudicated for MRC; self-report for all others.  Recorded as:   - Yes - No |
| Deep vein thrombosis | Form 30, Medical History  Form 33, Medical History Update (annual completion) | Since the date on the front of this form, have you been diagnosed or treated for any of the following conditions or procedures? (Blood clots in the veins of your legs [deep vein thrombosis or DVT])   - Yes - No | Any report of event on medical history update through date of supplement completion (2013) | Core study: Adjudicated for all.  Extension 1: Adjudicated for all.  Extension 2: Adjudicated for MRC; self-report for all others.  Used as recorded. |
| Pulmonary embolism | Form 30, Medical History  Form 33, Medical History Update (annual completion during extension 1 through 2010) | Since the date on the front of this form, have you been diagnosed or treated for any of the following conditions or procedures? (Blood clots in your lungs [pulmonary embolism or PE]   - Yes - No | Any report of event on medical history update through end of extension 1 (2010) | Core study: Adjudicated for all.  Extension 1: Adjudicated for all.  Extension 2: Adjudicated for MRC; self-report for all others.  Used as recorded. |
| History of hip fracture | Form 33, Medical History Update (annual completion) | Since the date on the front of this form, has a doctor or other health care provider told you that you have a new broken, fractured, or crushed hip or upper leg bone? IF YES, which bone(s) did you break, fracture, or crush? Mark all that apply:   - Hip | Any report of event on medical history update through date of supplement completion (2013) | Core study: Adjudicated for all.  Extension 1: Adjudicated for all.  Extension 2: Adjudicated for MRC; self-report for all others.  Used as recorded. |

Abbreviations: CT, clinical trial arm for core study and extension 1; MRC, full outcomes ascertainment and documentation conducted in 2 important subgroups of women: those in the hormone trials plus all African American and Hispanic participants; NA, not applicable; OS, observational study arm for core study and extension 1; SRC, the remaining Women’s Health Initiative Extension Study participants will be followed with outcomes ascertainment limited to self (or proxy) report or passive follow-up sources of information unless or until funding is obtained to collect their medical records.
