# Supplementary material for: Predictors of Influenza and Pneumococcal Vaccination Among Participants in the Women’s Health Initiative
Source: Public Health Rep. 2022 Mar 18;138(2):281–91. doi: 10.1177/00333549221081817 (PMC10031837; doi:10.1177/00333549221081817)
Supplement: sj-docx-2-phr-10.1177_00333549221081817 – Supplemental material for Predictors of Influenza and Pneumococcal Vaccination Among Participants in the Women’s Health Initiative [file sj-docx-2-phr-10.1177_00333549221081817.docx]

**Online-Only Supplementary Table 2.** Univariate models of predictors of influenza (n = 71 848) and pneumococcal pneumonia (n = 69 041) vaccination, Women’s Health Initiative participants aged ≥65 years who responded to questions on vaccination history, 2013

| **Characteristic** | **Influenza vaccination, odds ratio (95% CI)** | **Pneumonia vaccination, odds ratio (95% CI)** |
| --- | --- | --- |
| Age, y | 1.03 (1.03-1.04) | 1.04 (1.03-1.04) |
| Race and ethnicity | | |
| Non-Hispanic White | 1 [Reference] | 1 [Reference] |
| Non-Hispanic Black | 0.46 (0.43-0.50) | 0.43 (0.40-0.47) |
| Hispanic | 0.59 (0.53-0.66) | 0.52 (0.46-0.58) |
| American Indian/Alaska Native | 0.56 (0.41-0.76) | 0.69 (0.49-0.97) |
| Asian/Pacific Islander | 1.00 (0.86-1.15) | 0.85 (0.74-0.99) |
| Other | 0.57 (0.47-0.68) | 0.61 (0.50-0.74) |
| Education | | |
| Some high school or less | 0.55 (0.49-0.61) | 0.53 (0.47-0.60) |
| High school diploma/GED | 0.80 (0.76-0.85) | 0.77 (0.72-0.82) |
| School after high school | 0.77 (0.74-0.81) | 0.85 (0.81-0.89) |
| College degree or higher | 1 [Reference] | 1 [Reference] |
| Annual household income, $ | | |
| <10 000 | 1 [Reference] | 1 [Reference] |
| 10 000-34 999 | 1.40 (1.22-1.59) | 1.43 (1.24-1.65) |
| 35 000-74 999 | 1.69 (1.48-1.93) | 1.59 (1.38-1.83) |
| ≥75 000 | 2.10 (1.83-2.40) | 1.77 (1.54-2.05) |
| Urbanicity index | | |
| Urban or large rural city/town | 1 [Reference] | 1 [Reference] |
| Small rural town | 0.77 (0.68-0.87) | 0.77 (0.68-0.87) |
| Marital status | | |
| Never married | 0.77 (0.70-0.85) | 0.88 (0.79-0.98) |
| Divorced or separated | 0.63 (0.60-0.66) | 0.75 (0.70-0.79) |
| Widowed | 0.87 (0.82-0.93) | 0.89 (0.83-0.95) |
| Currently married | 1 [Reference] | 1 [Reference] |
| Marriage-like relationship | 0.66 (0.58-0.76) | 0.77 (0.66-0.89) |
| Smoking status | | |
| Current | 0.62 (0.55-0.70) | 0.67 (0.59-0.77) |
| Past | 1.15 (1.10-1.19) | 1.10 (1.05-1.15) |
| Never | 1 [Reference] | 1 [Reference] |
| Alcohol use, no. of times per week | | |
| Never | 1 [Reference] | 1 [Reference] |
| <1-4 | 1.19 (1.14-1.25) | 1.10 (1.05-1.15) |
| ≥5 | 1.43 (1.34-1.52) | 1.16 (1.09-1.24) |
| Exercise history, no. of days per week | | |
| 0 or 1 | 1 [Reference] | 1 [Reference] |
| 2 or 3 | 1.17 (1.11-1.23) | 1.07 (1.02-1.13) |
| ≥4 | 1.10 (1.04-1.18) | 1.03 (0.96-1.10) |
| Self-rated health | | |
| Excellent | 1 [Reference] | 1 [Reference] |
| Very good | 1.17 (1.10-1.24) | 1.23 (1.15-1.31) |
| Good | 1.35 (1.26-1.44) | 1.45 (1.35-1.55) |
| Fair | 1.43 (1.30-1.58) | 1.61 (1.45-1.78) |
| Poor | 1.26 (0.93-1.72) | 1.45 (1.03-2.04) |
| Use of internet for health information | | |
| Yes | 1.12 (1.07-1.16) | 1.29 (1.23-1.34) |
| No | 1 [Reference] | 1 [Reference] |
| Past pneumonia diagnosis | | |
| Yes | 1.32 (1.25-1.38) | 2.16 (2.04-2.29) |
| No | 1 [Reference] | 1 [Reference] |
| Health insurance | | |
| None | 0.42 (0.39-0.46) | 0.47 (0.43-0.51) |
| Medicaid | 0.65 (0.51-0.83) | 0.69 (0.53-0.89) |
| Private, Medicare, military, or other | 1 [Reference] | 1 [Reference] |
| **Chronic diseases** | | |
| History of cancer (any) | | |
| Yes | 1.31 (1.23-1.38) | 1.29 (1.21-1.37) |
| No | 1 [Reference] | 1 [Reference] |
| Alzheimer’s disease, Parkinson’s disease, or dementia | | |
| Yes | 1.04 (0.95-1.14) | 0.72 (0.66-0.79) |
| No | 1 [Reference] | 1 [Reference] |
| Asthma or emphysema | | |
| Yes | 1.18 (1.11-1.25) | 1.55 (1.45-1.67) |
| No | 1 [Reference] | 1 [Reference] |
| Diabetes, hypertension, or high cholesterol | | |
| Yes | 1.48 (1.42-1.55) | 1.43 (1.36-1.50) |
| No | 1 [Reference] | 1 [Reference] |
| Myocardial infarction, stroke, transient ischemic attack, deep vein thrombosis, or pulmonary embolism | | |
| Yes | 1.13 (1.07-1.20) | 1.24 (1.16-1.32) |
| No | 1 [Reference] | 1 [Reference] |
| History of hip fracture | | |
| Yes | 1.11 (0.98-1.27) | 1.29 (1.12-1.49) |
| No | 1 [Reference] | 1 [Reference] |

Abbreviation: GED, General Educational Development.
